# ROS-mediated TNF-α and MIP-2 gene expression in alveolar macrophages exposed to pine dust

**DOI:** 10.1186/1743-8977-1-3

**Published:** 2004-12-13

**Authors:** Huayan Long, Tingming Shi, Paul J Borm, Juha Määttä, Kirsti Husgafvel-Pursiainen, Kai Savolainen, Fritz Krombach

**Affiliations:** 1 Institute for Surgical Research, University of Munich, Munich, Germany; 2 Institut für Umweltmedizinische Forschung, University of Düsseldorf, Düsseldorf, Germany; 3 Department of Industrial Hygiene and Toxicology, Finnish Institute of Occupational Health, Helsinki, Finland

## Abstract

**Background:**

Respiratory symptoms, impaired lung function, and asthma have been reported in workers exposed to wood dust in a number of epidemiological studies. The underlying pathomechanisms, however, are not well understood. Here, we studied the effects of dust from pine (PD) and heat-treated pine (HPD) on the release of reactive oxygen species (ROS) and inflammatory mediators in rat alveolar macrophages.

**Methods:**

Tumour necrosis factor-alpha (TNF-α) and macrophage inflammatory protein-2 (MIP-2) protein release, TNF-α and MIP-2 mRNA expression, and generation of ROS were studied as end points after treatment of rat alveolar macrophages with PD or HPD. In a separate series of experiments, the antioxidants glutathione and N-acetyl-L-cysteine were included in combination with wood dust. To determine the endogenous oxidative and antioxidant capacity of wood dusts, electron spin resonance (ESR) spectroscopy was used.

**Results:**

After 4 h incubation, both PD and HPD elicited a significantly (p < 0.05) increased mRNA expression of TNF-α and MIP-2 as well as a concentration-dependent release of TNF-α and MIP-2 protein. Interestingly, PD induced a significantly higher TNF-α and MIP-2 production than HPD. Moreover, a significantly increased ROS production was observed in alveolar macrophages exposed to both PD and HPD. In the presence of the antioxidants glutathione and N-acetyl-L-cysteine, the PD- and HPD-induced release of ROS, TNF-α, and MIP-2 was significantly reduced. Finally, electron spin resonance analyses demonstrated a higher endogenous antioxidant capacity of HPD compared to PD. Endotoxin was not present in either dust sample.

**Conclusion:**

These results indicate that pine dust is able to induce expression of TNF-α and MIP-2 in rat alveolar macrophages by a mechanism that is, at least in part, mediated by ROS.

## Background

In addition to sino-nasal cancer [1], exposure to wood dust has been shown to be associated with a wide variety of acute and chronic non-malignant respiratory health effects as well as eye irritation and dermatitis [2,3]. However, the underlying mechanisms involved are not well understood and subject of controversial discussion. Although inflammatory markers were found in nasal and bronchoalveolar lavage fluid from wood-dust exposed individuals [4-6], other studies do not corroborate the hypothesis that inflammation plays a part in wood dust-induced airway obstruction [7]. Moreover, recent studies do not support the assumption that the complaints related to exposure to wood dust are IgE-mediated [8,9].

In wood processing facilities, the proportion of respirable wood dust ranges from 6% to 75% of the total wood aerosol [2]. Respirable wood dust particles may deposit in the pulmonary alveoli and interact with alveolar macrophages, a cell type that plays an important role in phagocytosis and clearance of inhaled particulates. Upon interaction with noxious particles, alveolar macrophages can produce a broad spectrum of pro-inflammatory mediators, such as tumour necrosis factor-alpha (TNF-α) and macrophage inflammatory protein-2 (MIP-2) as well as reactive oxygen (ROS) and nitrogen species [10-13]. TNF-α is one of the pre-eminent cytokines that acts as an initiator of inflammatory processes in the lung [14]. The chemokine MIP-2 is known to mediate neutrophilic inflammatory responses in the lung [10,15]. ROS have been shown not only to damage cells by peroxidizing lipids and disrupting DNA and proteins, but also to exert signaling functions and modulate gene transcription [16,17]. Moreover, ROS are suggested to mediate the release of TNF-α and MIP-2 in alveolar macrophages exposed to noxious particles [18]. Interestingly enough, a recent study demonstrated that exposure to pine dust induced increased ROS production and caused cell death in both murine RAW 264.7 macrophages and human polymorphonuclear leukocytes [19].

Pine is one of the most extensively used wood species in the wood processing industry and several studies have shown that exposure to pine dust induced respiratory symptoms, reduced lung function, and asthma [3,20-22]. Moreover, pine is one of the most common wood species used for heat treatment, one of the treatment processes for stabilization and preservation of wood. After heat-treatment, both physical and chemical properties of wood are changed [23].

This study aimed to investigate the effect of dust from untreated as well as from heat-treated pine on the production of TNF-α, MIP-2, and ROS by primary rat alveolar macrophages and to elucidate the role of oxidative stress in pine dust-induced cytokine production.

## Methods

### Wood dust

Dust from untreated pine (PD) and heat-treated pine (HPD) was obtained from the Kuopio Regional Institute of Occupational Health (Kuopio, Finland). Dusts were produced using a dust collecting face-grinding machine with 400-grit sanding paper. For particle size distribution analyses, wood dust specimens, gold-coated for 170 seconds with BAL-TEC SCD 005 Sputter Coater (BAL-TEC AG, Liechtenstein), were examined on a JEOL JSM-6400 scanning electron microscope (JEOL Inc., Peabody, MA) at an acceleration voltage of 20 kV. More than 1700 particles for each dust were analyzed from electron micrographs. More than 95% of wood dust particles from both pine and heat-treated pine had a diameter less than 5 μm (Table 1). The endotoxin content in PD and HPD as analyzed with a LAL gel-clot assay (Charles River, Germany) was below the detection limit of 0.06 EU/ml. For experiments, pine dust was suspended in RPMI-1640 medium with 10% fetal calf serum, ultrasonicated, and vortexed.

**Table 1:** Size distribution of wood dust particles

	Pine	Heat-treated Pine
Number of particles counted	1796	1928
< 1 μm (%)	66.9	65.6
1–5 μm (%)	29.6	30.0
5–10 μm (%)	3.1	3.3
10–20 μm (%)	0.4	0.8
20–50 μm (%)	0.1	0.1
> 50 μm (%)	0	0.1

### Collection of Alveolar Macrophages

Male Sprague-Dawley rats (Charles River, Sulzfeld, Germany) were anesthetized by an intraperitoneal injection of sodium pentobarbital (30 mg/KG body weight) and killed by exsanguination from the abdominal aorta. The lungs were lavaged ten times with 10 ml of sterile, non-pyrogenic phosphate-buffered saline solution (PBS; Serva, Heidelberg, Germany). The pooled samples were centrifuged at 300 g for 10 min, and the cell pellet was washed twice and re-suspended in RPMI 1640 (Seromed, Munich, Germany) supplemented with L-glutamine, gentamycin (0,16 mg/ml), and 10% heat-inactivated fetal bovine serum (FBS; Gibco BRL, Eggenstein, Germany). Total cell counts were assessed with a standard hemocytometer (Coulter Electronics, Krefeld, Germany). Air-dried cytocentrifuged smears served to identify the cellular populations after staining with May-Grünwald-Giemsa. The preparation of bronchoalveolar cells contained about 97–100% alveolar macrophages. Cell viability as determined by trypan blue exclusion was greater than 90%.

### Treatment of cells

Alveolar macrophages were adjusted according to the differential cell counts to 2 × 10^6 ^cells/ml. Then, 100 μl-samples of cell suspension were plated to 96-well flat-bottomed cell culture plates (Nunclon Delta, Roskilde, Denmark), and incubated at 37°C in 5% CO_2 _and 21% O_2_. After 2 h, non-adherent cells were removed by washing twice with RPMI 1640, and the adherent alveolar macrophages were covered with 100 μl of pine dust suspension at concentrations ranging from 5 to 200 μg/ml. As a negative control, 3-μm polystyrene microspheres (Polysciences, Eppelheim, Germany) were used at a concentration of 100 μg/ml. As a positive control, *Escherichia coli *LPS serotype 055:B5 purchased from Sigma Chemie (Taufkirchen, Germany) was used at a concentration of 100 ng/ml. In a separate series of experiments, alveolar macrophages were treated with 6 mM glutathione (GSH; Sigma-Aldrich, Steinheim, Germany) or 20 mM N-acetyl-cysteine (NAC; Sigma-Aldrich, Steinheim, Germany) for 30 min. Subsequently, the culture medium was replaced with 100 μl of pine dust suspension at a final concentration of 200 μg/ml. After 4 h incubation in the absence or presence of GSH (6 mM) or NAC (20 mM), supernatants were removed and stored at -20°C. There was no effect of either treatment on cell viability as measured by a LDH assay kit (Merck, Germany).

### RT-PCR

Total cellular RNA was extracted from pine dust-exposed alveolar macrophages using a ribonuclease protection kit (Rneasy Kit, QIAGEN, Hilden, Germany). RT-PCR was performed as described previously [24]. The oligonucleotide primers (MWG-Biotech, Ebersberg, Germany) used were 5'-TGC CTC AGC CTC TTC TCA TT-3' and 5'-TGT GGG TGA GGA GCA CAT AG-3' (EMBL: RNTNFAA, AC: X66539) for TNF, 5'-CAA TGC CTG ACG ACC CTA C-3' and 5'-CAG TTA GCC TTG CCT TTG TTC-3' [25] for MIP-2, and 5'-TCC CTC AAG ATT GTC AGC AA-3' and 5'AGA TCC ACA ACG GAT ACA TT-3' [26] for the housekeeping gene, glyceraldehyde-3-phosphate dehydrogenase (GAPDH). The sizes of the PCR products were 376 bp for TNF, 194 bp for MIP-2, and 309 bp for GAPDH. PCR products were visualized in 2% agarose gels containing 1% ethidium bromide. For densitometric analyses, BIO-1D V 96 software (Vilber Lourmat, Marne La Vallee, France) was used.

### TNF-α and MIP-2 ELISA

Concentrations of TNF-α and MIP-2 in culture supernatants were determined by enzyme-linked immunosorbent assay (ELISA) using commercially available kits (Biosource, Solingen, Germany).

### Detection of intracellular ROS

To detect intracellular ROS, 2',7'-dichlorofluorescin diacetate (DCFH-DA) (MoBiTec, Göttingen, Germany) was used. DCFH-DA diffuses into the cell and is hydrolyzed by intracellular esterases to polar 2',7'-dichlorofluorescin. This non-fluorescent fluorescin analogue can be oxidized to highly fluorescent 2',7'-dichlorofluorescein by intracellular oxidants [27]. Alveolar macrophages were cultured to adhere and incubated with 10 μM DCFH-DA for 30 min. The cultures were washed twice with RPMI 1640 and subsequently treated as described before. Baseline fluorescence was measured with a fluorometer (FLUOstar, BMG LabTechnologies, Offenburg, Germany) immediately after wood dusts were added. After 4 h of incubation under 37°C in 5% CO_2 _and 21% O_2_, fluorescence was measured again. The results are shown as percentage change from baseline values. The addition of 1 μM H_2_O_2 _served as an internal positive control.

### Electron spin resonance spectroscopy

Hydroxyl radical formation by wood dusts was assessed by electron spin resonance (ESR) spectroscopy, as described previously [28]. Briefly, wood dust suspensions (20 mg/ml) were prepared in pure water. 100 μl of this suspension was mixed with 200 μl of the spin trap 5,5-dimethyl-1-pyrroline-N-oxide (DMPO, 0.05 M in PBS) (Sigma, St. Louis, MO) and 100 μl H_2_O_2 _(0.5 M in PBS) (Fluka, Seelze, Germany). The suspension was incubated for 15 min at 37°C in a shaking water bath, and filtered through a 0.2 μm filter (15 mm syringe filter, Satorius AG, Goettingen, Germany) to remove particles from the suspension. The filtrate was immediately transferred to a capillary and measured with a Miniscope ESR spectrometer (Magnettech, Berlin, Germany). The antioxidant activity of wood dust suspensions was measured by using the stable spin label TEMPOL (Sigma, Steinheim, Germany). TEMPOL was added to wood dust suspensions (10 mg/ml) at a final concentration of 5 μM, mixed and incubated at 37°C for 1 hour in a shaking water bath. After filtering the suspension through a 0.2 μm filter, the filtrate was measured as mentioned above. ESR spectra were recorded at room temperature using the following instrumental conditions: Magnetic field: 3360 G, sweep width: 100 G, scan time: 30 sec, number of scans: 3, modulation amplitude: 1.8 G, receiver gain: 1000. Quantification was carried out as the sum of total amplitude on first derivation of ESR signal, and outcomes are expressed as the total amplitude in arbitrary units.

### Statistical analysis

Results are presented as mean ± SEM. Statistical comparisons were performed by using RM ANOVA with Student-Newman-Keuls method for multiple comparison procedures. A p value < 0.05 was considered significant.

## Results

### TNF-α and MIP-2 mRNA expression

**Figure 1:**
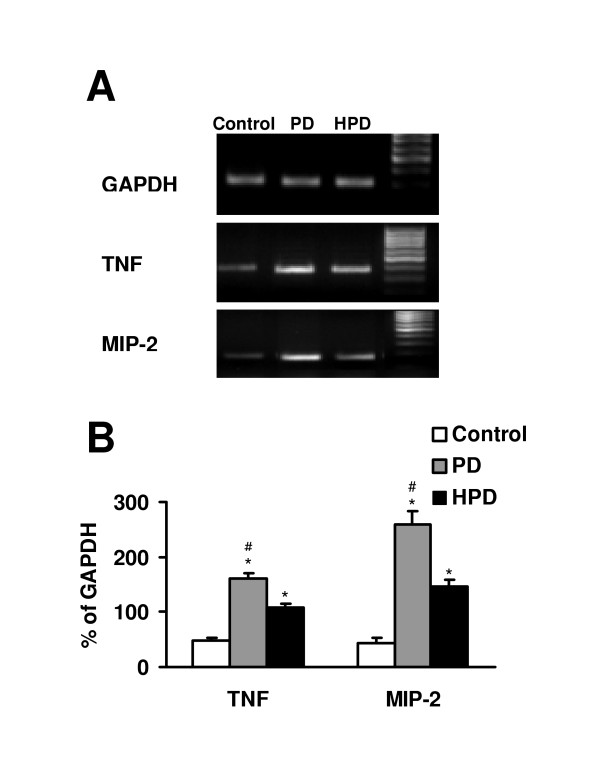
TNF-α and MIP-2 mRNA expression in alveolar macrophages after exposure to PD and HPD (100 μg/ml) for 4 h. (A) RT-PCR products of GAPDH, TNF-α and MIP-2 in an ethidium bromide stained agarose gel. Data shown are from four representative experiments. GAPDH were used as normalization control. (B) Densitometric analysis of 4 gels. *p < 0.05 compared with untreated control. #p < 0.05 compared with HPD.

After 4 h exposure of alveolar macrophages to PD and HPD, mRNA was extracted and the supernatants were collected for cytokine and chemokine measurement. A low, basal level of TNF-α and MIP-2 mRNA expression was observed in control macrophages. Compared to control, TNF-α and MIP-2 mRNA expression in alveolar macrophages exposed to PD and HPD was significantly increased. Interestingly, PD induced significantly (p < 0.05) higher levels of TNF-α and MIP-2 mRNA expression than HPD (Figure 1).

### TNF-α and MIP-2 release

**Figure 2:**
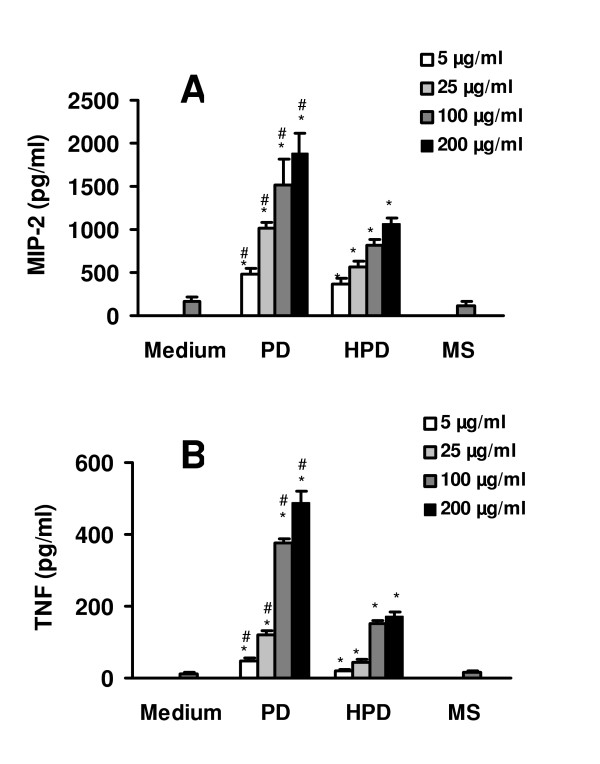
TNF-α and MIP-2 protein release after exposure of alveolar macrophages to PD, HPD, and polystyrene microspheres (MS) assayed with ELISA kits. Alveolar macrophages were incubated with 5, 25, 100, or 200 μg/ml dust for 4 h. (A) TNF-α release, (B) MIP-2-release. Values represent means ± SEM of six separate experiments performed in duplicate. *p < 0.05 compared with medium alone. #p < 0.05 compared with HPD.

As shown in Figure 2, exposure of alveolar macrophages to both PD and HPD elicited a significantly (p < 0.05) increased production of TNF-α and MIP-2 when compared to untreated control cells. This effect was concentration-dependent and already observed at the lowest concentration of 5 μg/ml. Moreover, PD induced a 1.3–2.8 fold (p < 0.05) higher release of TNF-α and MIP-2 than HPD. Polystyrene microspheres at a concentration of 100 μg/ml, which served as a negative control, did not induce an increased production of TNF-α and MIP-2. In contrast, exposure to LPS at a concentration of 100 ng/ml, which served as a positive control, induced a strong release of both mediators (data not shown).

### ROS generation

**Figure 3:**
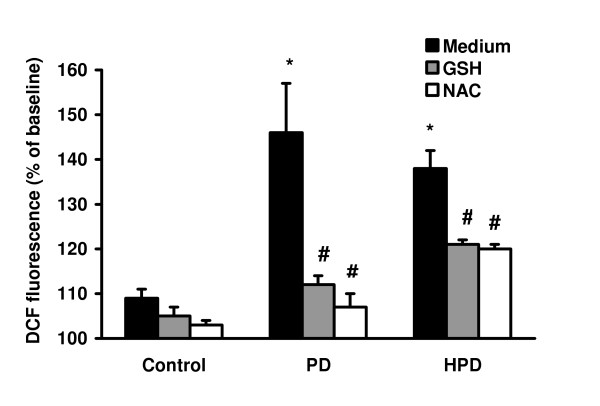
Production of intracellular ROS in alveolar macrophages after 4 h of stimulation with 200 μg/ml pine dust in the absence and presence of GSH (6 mM) or NAC (20 mM). Values are mean ± SEM of 6 experiments performed in duplicate. *p < 0.05 compared with untreated control. #p < 0.05 compared with PD or HPD without GSH or NAC treatment.

To detect ROS production in PD- and HPD-stimulated alveolar macrophages, the oxidant-sensitive dye DCFH-DA was used. After 4 h incubation, wood dusts at a concentration of 200 μg/ml induced a significantly (p < 0.05) increased ROS generation when compared to untreated control cells (Figure 3). However, the level of PD-induced ROS generation in alveolar macrophages was not statistically different from the level of HPD-induced ROS generation. Treatment of the cells with GSH (6 mM) or NAC (20 mM) caused significant suppression of both PD- and HPD-induced ROS generation.

### Effect of antioxidants on cytokine and chemokine expression

**Figure 4:**
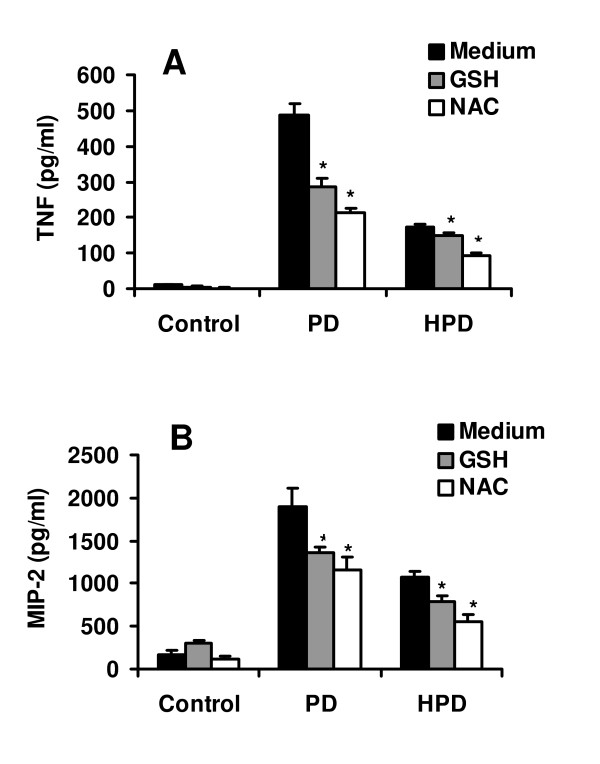
TNF-α and MIP-2 protein release of alveolar macrophages after 4 h of stimulation with 200 μg/ml pine dust in the absence and presence of GSH (6 mM) or NAC (20 mM). (A) TNF-α release, (B) MIP-2 release. Values represent means ± SEM of six separate experiments performed in duplicate. *p < 0.05 compared with PD or HPD without GSH or NAC treatment.

To elucidate whether oxidative stress participates in the up-regulation of inflammatory cytokine expression, TNF-α and MIP-2 release was examined in PD- and HPD-exposed alveolar macrophages in the presence or absence of the antioxidants GSH and NAC. Treatment with both GSH and NAC significantly (p < 0.05) reduced the TNF-α and MIP-2 release elicited by the exposure of alveolar macrophages to PD and HPD (Figure 4).

### Endogenous oxidant and antioxidant activity of pine dust

**Figure 5:**
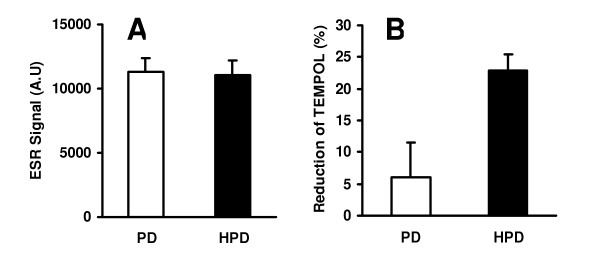
Hydroxyl radical formation (A) and antioxidant capacity (B) of PD and HPD as measured by ESR spectroscopy. Data are from three representative experiments.

ESR spectroscopy showed that suspensions of both PD and HPD caused formation of •OH in the presence of H_2_O_2_. However, the ability of PD and HPD to generate •OH was not statistically different (Figure 5). The antioxidant capacity of pine dust suspensions was measured by the use of the stable spin label TEMPOL. Interestingly, HPD caused a significantly greater reduction of TEMPOL than PD, indicating that HPD has greater antioxidant capacity than PD (Figure 5).

## Discussion

A higher prevalence of non-malignant respiratory diseases, such as bronchitis, chronic obstructive pulmonary disease, cryptogenic fibrosing alveolitis, and asthma has been reported in workers exposed to a variety of wood dusts [2]. Sensitization to wood dust from some wood species such as red cedar has been shown to be involved in mechanisms generating a work-related asthmatic response [29]. However, more recent studies have shown that sensitization to wood dust from pine, oak, beech and other wood species may not be the only or even the most important mechanism involved in wood dust-induced respiratory symptoms [8,9]. Therefore, our study aimed to investigate the non-specific inflammatory response of primary lung macrophages to wood dust from pine, one of the most extensively used wood species in the wood processing industry.

Here we show that pine dust induces TNF-α and MIP-2 mRNA expression as well as TNF-α and MIP-2 protein release in rat alveolar macrophages. Alveolar macrophages are important in processing airborne particles and play a key role in mediating inflammatory responses of the lung through the release of various proteolytic enzymes, reactive oxygen and nitrogen species, arachidonic acid metabolites, cytokines such as TNF-α, and chemokines such as MIP-2 [11]. TNF-α plays an important role as a mediator of the respiratory tract's response to particles. Studies have shown that a variety of agents which elicit marked lung inflammation can activate alveolar macrophages to release TNF-α, while agents with limited inflammatory activity do not stimulate macrophage TNF-α production. MIP-2 plays a major role in mediating the neutrophilic inflammatory response of the rodent lung to particles such as quartz and crocidolite asbestos [10]. TNF-α and MIP-2 gene expression is under the control of redox-sensitive inflammation-related transcription factors such as NF-κB. Activation of NF-κB is regulated via a number of second messengers, including calcium and ROS [16]. In addition to providing evidence that pine dust stimulates both TNF-α and MIP-2 mRNA production and protein release from rat alveolar macrophages, our study clearly demonstrates that pine dust stimulates the generation of ROS in alveolar macrophages, as previously shown in mouse macrophages and human leukocytes by Naarala et al. [19].

To investigated the role of oxidative stress in pine dust-induced cytokine and chemokine response we treated pine dust-exposed alveolar macrophages with the antioxidants GSH and NAC. GSH plays a major role in the antioxidant system by working as a substrate for glutathione peroxidase, and it has been previously shown that extracellular GSH can elevate intracellular GSH levels and protect phagocytes against oxidant damage [30]. NAC is a thiol compound that can act as a cysteine source for the repletion of intracellular glutathione and act as a direct scavenger of ROS. NAC has been shown to attenuate oxidant-mediated toxicity induced by chrysotile fibres in rats [31] and to down-regulate the nitric oxide pathway in alveolar macrophages [22]. We found that treatment with GSH or NAC attenuated pine dust-induced ROS generation as well as TNF-α and MIP-2 protein release. These findings are concordant with previous studies on silica and ultrafine particles [18,33,34] and indicate that pine dust-induced oxidative stress mediates, at least in part, the expression of TNF-α and MIP-2 in alveolar macrophages.

Interestingly enough, dust from untreated pine (PD) induced a significantly stronger inflammatory response in alveolar macrophages than dust from heat-treated pine (HPD). Consequently, we used ESR spectroscopy to assess the endogenous oxidative and antioxidant capacity of the wood dusts under study. Whereas the ability to generate hydroxyl radical did not differ among PD and HPD, HPD exhibited greater antioxidant capacity than PD. As we have demonstrated in this study that oxidative stress may play a role in mediating the expression of TNF-α and MIP-2, we suggest that the greater antioxidant capacity of HPD may neutralize oxidative stress and thus attenuate expression of TNF-α and MIP-2. As mentioned before, both physical and chemical properties of wood are changed when heat-treated for several hours with temperatures up to 230°C. In particular, the pine resin is easily volatilized and almost completely removed from the wood [23]. One of the components of resin, δ-3-carene, has been reported to decrease the viability of alveolar macrophages and affect the engulfment of particles *in vitro *[35]. Another component of pine resin, abietic acid, has been shown to produce lytic damage to alveolar, tracheal, and bronchial epithelial cells [36]. Further studies are warranted to confirm our results and to determine the specific chemical and physical properties of dust from heat-treated pine that might be responsible for the effects seen in this study. Recently, metabolites of pine bark extract have been shown to have antioxidant activity and to inhibit matrix metalloproteinases (37).

In summary, our findings indicate that non-specific inflammatory reactions, mediated *via *ROS production, may play a role in pulmonary effects of wood dust. However, it is not clear from this *in vitro *study whether the oxidative stress driving TNF-α and MIP-2 protein release is due to ROS derived directly from the dust particles or from cell-generated ROS.

## Conclusions

Here, we demonstrate that pine dust is able to induce inflammatory responses *in vitro*. Oxidative stress seems to play an important role in the pine dust-induced cytokine and chemokine response, suggesting that wood dust particles may exert pro-inflammatory effects by a mechanism that is, at least in part, mediated by ROS.

## Authors' contributions

HL performed the isolation of alveolar macrophages, subsequent cytological and biochemical analyses, and writing and preparation of the manuscript. TS and PJB carried out ESR spectroscopy. JM analysed the size distribution of wood dust particles. KHP, KS, and FK participated in the direction of the study as well as in writing and preparation of the manuscript. All authors read and approved the final manuscript.
